# A numerical exploration of the comparative analysis on water and kerosene oil-based Cu–CuO/hybrid nanofluid flows over a convectively heated surface

**DOI:** 10.1038/s41598-024-53024-0

**Published:** 2024-01-30

**Authors:** Ebrahem A. Algehyne, Fahad Maqbul Alamrani, Anwar Saeed, Gabriella Bognár

**Affiliations:** 1 Department of Mathematics, Faculty of Science, University of Tabuk, P.O. Box 741, 71491 Tabuk, Saudi Arabia; 2 Nanotechnology Research Unit (NRU), University of Tabuk, 71491 Tabuk, Saudi Arabia; 3 Department of Mathematics, Abdul Wali Khan University, Mardan, Khyber Pakhtunkhwa 23200 Pakistan; 4 Institute of Machine and Product Design, University of Miskolc, Miskolc-Egyetemváros, 3515 Hungary

**Keywords:** Engineering, Mathematics and computing

## Abstract

The fluid flow over an extending sheet has many applications in different fields which include, manufacturing processes, coating, thin film decomposition, heat and mass transfer, biomedical applications, aerospace engineering, environmental science, energy production. Keeping in mind these applications, the non-Newtonian hybrid nanofluid flow comprising of Cu and CuO nanoparticles over an extending sheet is analyzed in this work. Two different base fluids called kerosene oil and water have been incorporated. The sheet is considered to be thermally convective along with zero mass flux condition. The main equations of modeled problem have been transformed to dimensionless form by using similarity variables. The designed problem is evaluated computationally by using bvp4c Matlab function. Validation of the present results is also performed. The impacts of magnetic, Brownian motion, chemical reaction, suction and thermophoresis factors are analyzed and discussed in details. The outcomes of the present investigation declare that the kerosene oil-based hybrid nanofluid flow has greater velocity and concentration profiles than that of the water-based hybrid nanofluid flow. The water-based hybrid nanofluid has greater temperature distribution than that of kerosene oil-based hybrid nanofluid flow. The streamlines of the kerosene oil-based Newtonian and non-Newtonian hybrid nanofluid flows are more stretched than water-based Newtonian and non-Newtonian hybrid nanofluid flows.

## Introduction

Nanofluid is a specialized type of fluid engineered by dispersing nanometer-sized particles, typically nanoparticles, within a pure fluid. These nanoparticles are often metallic or non-metallic and can include materials like metal oxides, carbon nanotubes, or graphene. When mixed with the base fluid as initially done by Choi and Eastman^1^, nanofluids exhibit unique and enhanced properties compared to the base fluid alone. Nanofluid flow is a significant area of research and application in fields like fluid dynamics, heat transfer, and materials science^2^. In nanofluid flow, researchers study how these nanoparticles interact with the base fluid and affect its thermal flow behavior^3^. The nanoscale additives significantly boost the overall heat transfer efficiency of the fluid, making nanofluids valuable in various industries, like electronics cooling, renewable energy systems, and industrial processes^4^. The improved heat transfer properties are particularly beneficial in forced convection, natural convection, boiling, and phase change applications, as nanofluids exhibit increased rates of heat dissipation and enhanced performance, though challenges related to nanoparticle dispersion and stability continue to be addressed for broader and more reliable implementation. Khan et al.^5^ scrutinized thermally the nanofluid flow in a conduit using the impacts of microorganisms and have noted that with growth in volumetric fraction of nanoparticles the thermal panels of fluid has augmented while flow distribution declined. Acharya et al.^6^ discussed variations in the thermal properties for nanofluid flow using the impacts of nano-layers and diameter of nanoparticles. Shahid et al.^7^ inspected experimentally the nanofluid flow with impacts of activated energy on a permeable surface. Bhatti et al.^8^ discovered MHD nanofluid flow with influence of microorganisms’ swimming through circular disks placed in porous medium and have noted that velocity distribution has declined growth in magnetic factor.

A hybrid nanofluid is an advanced engineered fluid consisting of a base fluid, typically a liquid, in which multiple types or sizes of nanoparticles are uniformly dispersed^9^. These nanoparticles can include various materials, such as metal oxides, carbon nanotubes, or graphene, and their combination within the fluid creates a multifunctional nanofluid with tailored thermal, electrical, or rheological properties^10^. Hybrid nanofluids find applications in diverse fields, including solar thermal system and electronics cooling where their unique composition synergistically enhances heat transfer efficiency and performance, offering a versatile solution to optimize thermal management in various industries^11,12^. Researchers continually explore the complex dynamics of heat transfer within hybrid nanofluids to harness their full potential for advanced thermal management solutions across various industries. Zhang et al.^13^ discussed hybrid nanofluid flow on a surface with nickel and tantalum nanoparticles using the impacts of magnetic field. Wang et al.^14^ simulated hybrid nanoparticles fluid flow in a microchannel by taking permeable media to analyze stability of CPU. Gumber et al.^15^ discussed hybrid nanofluid flow on a surface using the impacts of thermal radiations, injection / suction and micro-polarity effects. Ojjela^16^ investigated computationally the thermal transportation using silica-alumina hybrid nanofluid flow on an elongating sheet. Hassan et al.^17^ inspected experimentally the flow pattern for mass and thermal transportations for shear thinning hybrid-nanoparticles flow. The combination of nanoparticles within the hybrid nanofluid imparts tailored and synergistic thermal properties, including enhanced thermal conductivity and heat transfer efficiency^18^. This enables the fluid to effectively dissipate and manage heat in industrial engineering thereby contributing to improved energy efficiency and system performance^19^. Raizah et al.^20^ examine hybrid nanofluid flow within a gyrating channel, with impression of chemically reactivity processes governed by Arrhenius activated energy and proved that thermal distribution has escalated with escalation in nanoparticles’ concentration. Further related articles can be read in Refs.^21–27^.

Magnetohydrodynamic (MHD), is a field of physics and engineering consisting of electrically conducting fluids, in the presence of magnetic and electric fields^28^. In MHD, the fluid's motion and electromagnetic properties are strongly coupled, leading to various interesting phenomena and applications^29^. MHD has wide-ranging applications, including in astrophysics (like studying stars and galaxies), nuclear fusion research (for controlled fusion reactions)^30^. Heat transmission in MHD fluid flow explores the collaboration between the flow of electrically conducting fluids using magnetic fields^31^. In this complex system, the magnetic field profoundly influences fluid behavior, altering heat transfer mechanisms. MHD-induced currents generate Joule heating, impacting heat transfer rates, while the magnetic field itself can suppress turbulence and shape flow patterns. Additionally, MHD fluid flow considerations are relevant in engineering applications like induction heating and heat shielding during spacecraft re-entry, making it a crucial field at the intersection of fluid dynamics, electromagnetism, and heat transfer. Kumawat et al.^32^ examined production of entropy and thermal transportation for MHD flow in a permeable artery with impacts of deviated viscosity and have noted that curvature of arterial wall contribute to an increased hazard of atherosclerosis development, whereas the presence of a heat source within the bloodstream has appeared to reduce this risk. Asjad et al.^33^ studied influences of MHD on Williamson liquid flow using bio-convective effects. Bejawada et al.^34^ inspected radiative effects on MHD flow of fluid on a sheet with chemical reactivity using Darcy-Forchheimer permeable medium.

Heat transfer in fluid flow involving Brownian motion and thermophoresis is a complex interaction of several phenomena^35^. In Brownian motion, the haphazard movement of mixed particles due to collisions with fluid molecules contributes to the dispersion of heat-absorbing or heat-emitting particles within the fluid. This dispersion affects the local temperature distribution as particles move erratically. Similarly, thermophoresis, driven by temperature gradients, causes particles to migrate toward regions of higher or lower temperature depending on their thermal properties, thus influencing the heat transfer process. Together, these mechanisms impact the effective thermal conductivity and heat distribution in the fluid, making them crucial considerations in various applications^36^. Brownian motion and thermophoresis play significant roles in various applications within fluid flow^37^. In aerosol science and environmental engineering, Brownian motion is crucial for understanding the dispersion and transport of small particles, such as pollutants or aerosols, in the atmosphere. It influences particle size distributions and can affect air quality and health outcomes. Thermophoresis, on the other hand, finds applications in areas like particle separation and manipulation within microfluidic devices. By exploiting the thermal properties of particles, thermophoresis can be used to sort and control the movement of particles in microscale systems, facilitating tasks such as DNA sequencing and drug delivery. These phenomena have diverse applications, from nanotechnology and pharmaceuticals to environmental monitoring and advanced materials research, ultimately impacting a wide range of industries and scientific disciplines^38^. Waqas et al.^39^ debated on computationally the impacts of thermophoresis and Brownian motion on rotational fluid flow on a rectangular plate with permeability effects. Sharma et al.^40^ inspected water flow conveying nanoparticles on a gyrating disk with significances of thermophoresis and Brownian motion effects. Sulochana et al.^41^ studied the Joule heating impacts on MHD fluid flow on a surface with varying thickness using the influence of Brownian movement and thermophoresis along with no-slip and slip constraints.

The Maxwell hybrid nanofluid flow containing copper and copper oxide nanoparticles has direct significance to numerous problems from the engineering fields. In many engineering problems, the Maxwell hybrid nanofluids improve the heat transfer efficiency in systems like electronics devices, heat exchangers and automotive cooling systems. Such nanofluids can also improve the thermal properties of heat transfer fluids in solar collectors, heat absorption and dissipation, managing high temperature in engines. In summary, the analysis of such hybrid nanofluids provides the solutions to various engineering problems related to energy efficiency, heat transfer, material science and sustainable technologies.

From the above literature, the authors confirm that a comparison of water as well as kerosene oil-based hybrid nanofluid flows on a convectively heated sheet with zero mass flux constraints have not been performed yet. Therefore, the authors have intended to perform a comparative analysis on such a topic. Some external forces are also taken into consideration so that we can analyze the hybrid nanofluid flows against various emerging factors while with water and kerosene oil as pure fluids. Thus, the authors have planned the following research questions, which have to be determined by completing this analysis:

- While using water and kerosene oil as base fluids, which one of the hybrid nanofluids flows, will have higher velocity, temperature, and concentration profiles?
- What will be the streamlines of behavior for Newtonian and non-Newtonian hybrid nanofluid flows while using two different base fluids?
- For both magnetized and non-magnetized flows, what will be the streamlines behavior of a hybrid nanofluid flow while using two different base fluids?

To answer these research questions, the authors have formulated a mathematical model in Section "Model formulation" with its computational solution in Section "Numerical procedure". Validation of the modeled problem is given in Section "Validation" with discussion of the main results in Section "Discussion of Results". Section "Conclusion" is used to conclude the problem.

## Model formulation

**Figure 1:**
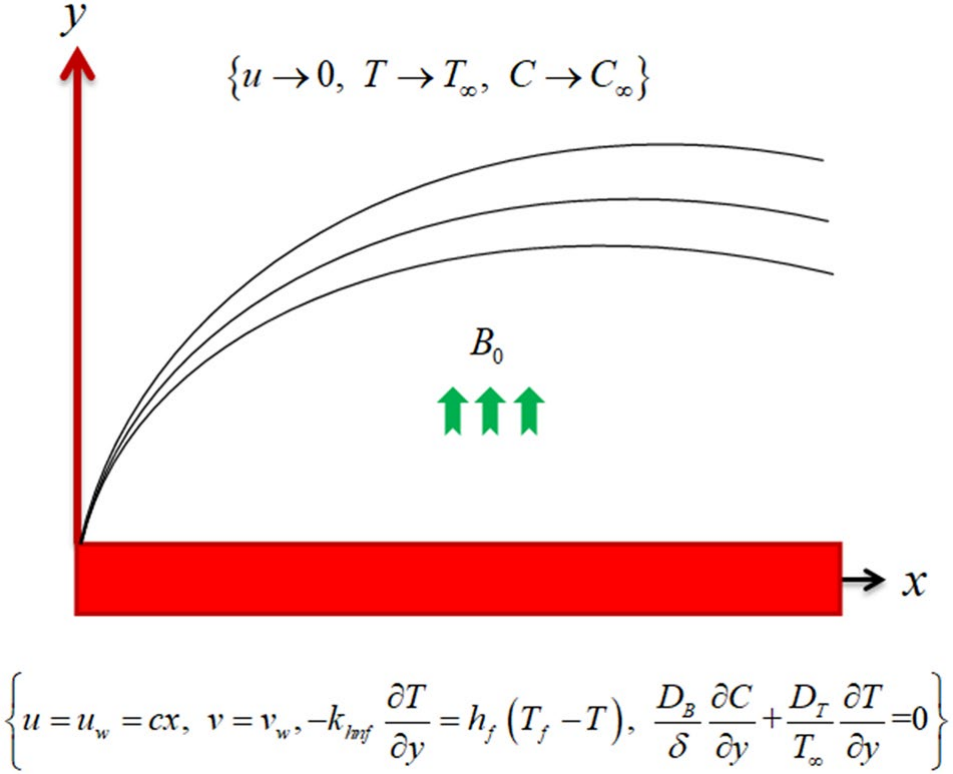
Graphical view of flow problem.

Take two-dimensional flow of Maxwell hybrid nanofluid on a stretching sheet. The hybrid nanofluid flow is composed of two different nanoparticles called copper and copper oxide with pure fluids as water and kerosene oil. The sheet stretches along *x*-axis with velocity $u_{w} = cx$ and *y*-axis as normal to fluid flow. $B_{0}$ is potential of magnetic field taken normal to fluid flow as depicted in Fig. 1. It is considered that the surface is convective such that $T_{f} > T_{w} > T_{\infty }$ along with zero mass flux condition ($C_{w} = 0$). Here, $T_{f}$ shows temperature of hot working fluid which transfers heat with heat transfer coefficient $h_{f}$, $T_{w}$ and $T_{\infty }$ are surface as well as ambient temperatures. Also, $C_{w}$ and $C_{\infty }$ are surface and ambient concentrations. The impacts of magnetic field, viscous dissipation, thermophoresis, chemical reaction, Brownian motion are used in order to investigate the hybrid nanofluid flows profiles. Thus, the leading equations are^42–44^:

1 $$ \frac{\partial u}{{\partial x}} + \frac{\partial v}{{\partial y}} = 0, $$

2 $$ v\,\,\frac{\partial \,u}{{\partial y}} + u\,\,\frac{\partial \,u}{{\partial x}} = \frac{{\mu_{hnf} }}{{\rho_{hnf} }}\frac{{\partial^{2} u}}{{\partial y^{2} }} - \lambda \left[ {u^{2} \frac{{\partial^{2} u}}{{\partial x^{2} }} - 2uv\frac{{\partial^{2} u}}{\partial x\partial y} + v^{2} \frac{{\partial^{2} u}}{{\partial y^{2} }}} \right] - \frac{{\sigma_{hnf} B_{0}^{2} }}{{\rho_{hnf} }}\left( {\lambda v\frac{\partial u}{{\partial y}} + u} \right), $$

3 $$ v\,\,\frac{\partial \,T}{{\partial \,y}} + u\,\,\frac{\partial \,\,T}{{\partial \,x}} = \frac{{k_{hnf} }}{{\left( {\rho C_{p} } \right)_{hnf} }}\frac{{\partial^{2} T}}{{\partial y^{2} }} + \frac{{\mu_{hnf} }}{{\left( {\rho C_{p} } \right)_{hnf} }}\left( {\frac{\partial u}{{\partial y}}} \right)^{2} + \frac{{\left( {\rho C_{p} } \right)_{np} }}{{\left( {\rho C_{p} } \right)_{hnf} }}\left( {\frac{{D_{B} }}{\delta }\frac{\partial T}{{\partial y}}\frac{\partial C}{{\partial y}} + \frac{{D_{T} }}{{T_{\infty } }}\left( {\frac{\partial T}{{\partial y}}} \right)^{2} } \right), $$

4 $$ v\,\,\frac{\partial \,C}{{\partial \,y}} + u\,\frac{\partial \,C}{{\partial \,x}} = D_{B} \frac{{\delta D_{T} }}{{T_{\infty } }}\frac{{\partial^{2} C}}{{\partial y^{2} }}\frac{{\partial^{2} T}}{{\partial y^{2} }} - Kr\left( {C - C_{\infty } } \right), $$

Constraints at boundaries are defined as^45^:

5 $$ \left\{ \begin{gathered} u = u_{w} = cx,\,\,\,v = v_{w} , - k_{hnf} \frac{\partial T}{{\partial y}} = \left( {T_{f} - T} \right)h_{f} ,\,\,\,\frac{{D_{T} }}{{T_{\infty } }}\frac{\partial T}{{\partial y}}{ + }\frac{{D_{B} }}{\delta }\frac{\partial C}{{\partial y}}{ = 0}\,\,\,{\text{as}}\,\,\,y = 0, \hfill \\ \,\,\,\,\,\,\,\,\,\,\,\,\,\,\,\,\,\,\,\,\,\,\,\,\,\,\,\,\,\,\,u \to 0,\,\,\,\,\,\,C \to C_{\infty } ,\,\,T \to T_{\infty } \,\,as\,\,\,y \to \infty . \hfill \\ \end{gathered} \right\} $$

The thermophysical relations are defined as^46^:

6 $$ \left\{ \begin{gathered} \mu_{hnf} = \frac{{\mu_{f} }}{{\left( {1 - \varphi_{{p_{1} }} } \right)^{2.5} \left( {1 - \varphi_{{p_{2} }} } \right)^{2.5} }},\,\,\,\rho_{hnf} = \left( {1 - \varphi_{{p_{2} }} } \right)\left\{ {\rho_{f} \left( {1 - \varphi_{{p_{1} }} } \right) + \rho_{{p_{1} }} \varphi_{{p_{1} }} } \right\} + \varphi_{{p_{2} }} \rho_{{_{{_{{p_{2} }} }} }} , \hfill \\ \,\,\,\,\,\,\,\,\,\,\,\left( {\rho C_{p} } \right)_{hnf} = \left\{ {\left( {\rho C_{p} } \right)_{f} \left( {1 - \varphi_{{p_{1} }} } \right) + \left( {\rho C_{p} } \right)_{{_{{p_{1} }} }} \varphi_{{p_{1} }} } \right\}\left( {1 - \varphi_{{p_{2} }} } \right) + \varphi_{{p_{2} }} \left( {\rho C_{p} } \right)_{{_{{p_{2} }} }} ,\,\,\, \hfill \\ \,\,\,\,\,\,\,\,\,\,\frac{{k_{nf} }}{{k_{f} }} = \frac{{k_{{_{{p_{1} }} }} + 2k_{f} - 2\varphi_{{p_{1} }} \left( {k_{f} - k_{{_{{p_{1} }} }} } \right)}}{{k_{{_{{p_{1} }} }} + 2k_{f} + \varphi_{{p_{1} }} \left( {k_{f} - k_{{_{{p_{1} }} }} } \right)}},\,\,\frac{{k_{hnf} }}{{k_{nf} }} = \frac{{k_{{_{{p_{2} }} }} + 2k_{f} - 2\varphi_{{p_{2} }} \left( {k_{f} - k_{{p_{2} }} } \right)}}{{k_{{p_{2} }} + 2k_{f} + \varphi_{{p_{2} }} \left( {k_{f} - k_{{p_{2} }} } \right)}}, \hfill \\ \,\,\,\,\,\,\,\frac{{\sigma_{nf} }}{{\sigma_{f} }} = \frac{{\sigma_{{p_{1} }} + 2\sigma_{f} - 2\left( {\sigma_{f} - \sigma_{{p_{1} }} } \right)\varphi_{{p_{1} }} }}{{\sigma_{{p_{1} }} + 2\sigma_{f} + \left( {\sigma_{f} - \sigma_{{p_{1} }} } \right)\varphi_{{p_{1} }} }},\,\,\,\frac{{\sigma_{hnf} }}{{\sigma_{nf} }} = \frac{{\sigma_{{p_{2} }} + 2\sigma_{f} - 2\varphi_{{p_{2} }} \left( {\sigma_{f} - \sigma_{{p_{2} }} } \right)}}{{\sigma_{{p_{2} }} + 2\sigma_{f} + \varphi_{{p_{2} }} \left( {\sigma_{f} - \sigma_{{p_{2} }} } \right)}}. \hfill \\ \end{gathered} \right\} $$

The thermophysical characteristics of both the base fluids and nanomaterials can be found in Table 1.

**Table 1:** Thermophysical features of the water, kerosene oil, Cu and CuO^46,48^.

Properties	Water	Kerosene oil	$Cu$	$CuO$
$\rho$ $\left( {{\text{kg}}/{\text{m}}^{3} } \right)$	997.1	783	8933	6500
$C_{p}$ $\left( {{\text{J}}/{\text{kgK}}} \right)$	4179	2090	385	540
$k$ $\left( {{\text{W}}/{\text{mK}}} \right)$	0.613	0.15	401	18
$\sigma$ $\left( {{\text{S}}/{\text{m}}} \right)$	5.5 × 10^–5^	21 × 10^–6^	5.96 × 10^7^	2.7 × 10^–8^

Table 1 shows the thermophysical features of the water, kerosene oil,$Cu$ and $CuO$.

Variables used for similarity are:

7 $$ u = f^{\prime}\left( \eta \right)cx,\,\,\,v = - f\left( \eta \right)\sqrt {\nu_{f} c} ,\,\,\,\phi \left( \eta \right) = \frac{{C - C_{\infty } }}{{C_{\infty } }},\,\,\,\theta \left( \eta \right) = \frac{{T - T_{\infty } }}{{T_{f} - T_{\infty } }},\,\,\,\,\,\eta = y\sqrt {\frac{c}{{v_{f} }}} . $$

Using these similarity variables, Eq. (1) satisfies and Eqs. (2–5) are:

8 $$ \begin{aligned} & \frac{{{{\mu_{hnf} } \mathord{\left/ {\vphantom {{\mu_{hnf} } {\mu_{f} }}} \right. \kern-0pt} {\mu_{f} }}}}{{{{\rho_{hnf} } \mathord{\left/ {\vphantom {{\rho_{hnf} } {\rho_{f} }}} \right. \kern-0pt} {\rho_{f} }}}}f^{\prime\prime\prime}\left( \eta \right) + \frac{{{{\sigma_{hnf} } \mathord{\left/ {\vphantom {{\sigma_{hnf} } {\sigma_{f} }}} \right. \kern-0pt} {\sigma_{f} }}}}{{{{\rho_{hnf} } \mathord{\left/ {\vphantom {{\rho_{hnf} } {\rho_{f} }}} \right. \kern-0pt} {\rho_{f} }}}}M\left( {\beta f\left( \eta \right)f^{\prime\prime}\left( \eta \right) - f^{\prime}\left( \eta \right)} \right) - f^{{\prime}{2}} \left( \eta \right) \\ & \quad \, + f^{\prime\prime}\left( \eta \right)f\left( \eta \right) + \beta \left( {f^{2} \left( \eta \right)f^{\prime\prime\prime}\left( \eta \right) - 2f\left( \eta \right)f^{\prime}\left( \eta \right)f^{\prime\prime}\left( \eta \right)} \right) = 0, \\ \end{aligned} $$

9 $$ \begin{aligned} \, & \,\frac{{{{k_{hnf} } \mathord{\left/ {\vphantom {{k_{hnf} } {k_{f} }}} \right. \kern-0pt} {k_{f} }}}}{{{{\left( {\rho C_{p} } \right)_{hnf} } \mathord{\left/ {\vphantom {{\left( {\rho C_{p} } \right)_{hnf} } {\left( {\rho C_{p} } \right)_{f} }}} \right. \kern-0pt} {\left( {\rho C_{p} } \right)_{f} }}}}\theta^{\prime\prime}\left( \eta \right) + \frac{{{{\mu_{hnf} } \mathord{\left/ {\vphantom {{\mu_{hnf} } {\mu_{f} }}} \right. \kern-0pt} {\mu_{f} }}}}{{{{\left( {\rho C_{p} } \right)_{hnf} } \mathord{\left/ {\vphantom {{\left( {\rho C_{p} } \right)_{hnf} } {\left( {\rho C_{p} } \right)_{f} }}} \right. \kern-0pt} {\left( {\rho C_{p} } \right)_{f} }}}}\Pr Ecf^{{\prime\prime}{2}} \left( \eta \right)  \\ &\quad+ \Pr f\left( \eta \right)\theta^{\prime}\left( \eta \right) + \frac{\Pr }{{{{\left( {\rho C_{p} } \right)_{hnf} } \mathord{\left/ {\vphantom {{\left( {\rho C_{p} } \right)_{hnf} } {\left( {\rho C_{p} } \right)_{f} }}} \right. \kern-0pt} {\left( {\rho C_{p} } \right)_{f} }}}}\left( {Nb\theta^{\prime}\left( \eta \right)\phi^{\prime}\left( \eta \right) + Nt\theta^{{\prime}{2}} \left( \eta \right)} \right) = 0, \\ \end{aligned} $$

10 $$ \phi^{\prime\prime}\left( \eta \right) + Scf\left( \eta \right)\phi^{\prime}\left( \eta \right) + \frac{Nt}{{Nt}}\theta^{\prime\prime}\left( \eta \right) - K_{r} Sc\phi \left( \eta \right) = 0, $$

11 $$ \left\{ \begin{aligned} & \,\,\,f\left( 0 \right) = S,\,\,\,\,f^{\prime}\left( 0 \right) = 1,\,\,\,\,f^{\prime}\left( \infty \right) = 0, \\ & \frac{{k_{hnf} }}{{k_{f} }}\theta^{\prime}\left( 0 \right) = Bi\left( {\theta \left( 0 \right) - 1} \right),\,\,\,\,\theta \left( \infty \right) = 0, \\ \, & \,Nb\phi^{\prime}\left( 0 \right) + Nt\theta^{\prime}\left( 0 \right) = 0,\,\,\,\,\phi \left( \infty \right) = 0. \\ \end{aligned} \right\} $$

In Eqs. (8)–(11), the dimensionless parameters are defined as:

12 $$ \left\{ \begin{aligned} & M = \frac{{\sigma_{f} B_{0}^{2} }}{{c\rho_{f} }},\,\,\,Ec = \frac{{u_{w}^{2} }}{{\left( {C_{p} } \right)_{f} \left( {T_{f} - T_{\infty } } \right)}},\,\,\,\beta = \lambda c,\,\,\,\Pr = \frac{{\mu_{f} \left( {C_{p} } \right)_{f} }}{{k_{f} }},\,\,\,Sc = \frac{{\nu_{f} }}{{D_{B} }},\,\,\,\,K_{r} = \frac{Kr}{c}, \\ & Nt = \frac{{\left( {\rho C_{p} } \right)_{np} }}{{\left( {\rho C_{p} } \right)_{f} }}\frac{{D_{T} \left( {T_{f} - T_{\infty } } \right)}}{{\nu_{f} T_{\infty } }},\,\,\,\,\,Nb = \frac{{\left( {\rho C_{p} } \right)_{np} }}{{\left( {\rho C_{p} } \right)_{f} }}\frac{{D_{B} C_{\infty } }}{{\delta \nu_{f} }},\,\,\,\,\,\,Bi = \frac{{h_{f} }}{{k_{f} }}\sqrt {\frac{{\nu_{f} }}{c}} ,\,\,\,\,\,\,S = - \frac{{v_{w} }}{{\sqrt {c\nu_{f} } }}. \\ \end{aligned} \right\} $$

Skin friction, Nusselt and Sherwood numbers are described as:

13 $$ C_{fx} = \frac{{\tau_{wx} }}{{\rho_{f} u_{w}^{2} }},\,\,\,Nu_{x} = \frac{{xq_{w} }}{{k_{f} \left( {T_{f} - T_{\infty } } \right)}},\,\,\,Sh_{x} = \frac{{xq_{m} }}{{D_{B} \left( {C_{w} - C_{\infty } } \right)}}, $$

where

14 $$ \tau_{w} = \mu_{hnf} \left. {\frac{\partial u}{{\partial y}}} \right|_{y = 0} ,\,\,\,q_{w} = - k_{hnf} \left. {\frac{\partial T}{{\partial y}}} \right|_{y = 0} ,\,\,\,q_{m} = - D_{B} \left. {\frac{\partial C}{{\partial y}}} \right|_{y = 0} . $$

With use of Eq. (7), Eq. (13) converted to:

15 $$ {\text{Re}}_{x}^{1/2} C_{fx} = \frac{{\mu_{hnf} }}{{\mu_{f} }}f^{\prime\prime}\left( 0 \right),\,\,\,{\text{Re}}_{x}^{ - 1/2} Nu_{x} = - \frac{{k_{hnf} }}{{k_{f} }}\theta^{\prime}\left( 0 \right). $$

Here, the Sherwood number eliminates due to fact that the surface concentration is zero^45,47,49^.

## Numerical procedure

Take

16 $$ \left\{ \begin{aligned} & f\left( \eta \right) = \xi \left( 1 \right),\,\,\,f^{\prime\prime}\left( \eta \right) = \xi \left( 3 \right),\,f^{\prime}\left( \eta \right) = \xi \left( 2 \right),\,\,\,f^{\prime\prime\prime}\left( \eta \right) = \xi^{\prime}\left( 3 \right), \\ \, & \quad \theta \left( \eta \right) = \xi \left( 4 \right),\,\,\,\theta^{\prime}\left( \eta \right) = \xi \left( 5 \right),\,\,\,\theta^{\prime\prime}\left( \eta \right) = \xi^{\prime}\left( 5 \right), \\ & \,\quad \phi \left( \eta \right) = \xi \left( 6 \right),\,\,\,\phi^{\prime}\left( \eta \right) = \xi \left( 7 \right),\,\,\,\phi^{\prime\prime}\left( \eta \right) = \xi^{\prime}\left( 7 \right). \\ \end{aligned} \right\} $$

then

17 $$ \xi^{\prime}\left( 3 \right) = - \frac{{\left\{ {\frac{{{{\sigma_{hnf} } \mathord{\left/ {\vphantom {{\sigma_{hnf} } {\sigma_{f} }}} \right. \kern-0pt} {\sigma_{f} }}}}{{{{\rho_{hnf} } \mathord{\left/ {\vphantom {{\rho_{hnf} } {\rho_{f} }}} \right. \kern-0pt} {\rho_{f} }}}}M\left( {\beta \xi \left( 1 \right)\xi \left( 3 \right) - \xi \left( 2 \right)} \right) - \xi^{2} \left( 2 \right) + \xi \left( 1 \right)\xi \left( 3 \right) + \left( { - 2\beta \xi \left( 1 \right)\xi \left( 2 \right)\xi \left( 3 \right)} \right)} \right\}}}{{\left( {\frac{{{{\mu_{hnf} } \mathord{\left/ {\vphantom {{\mu_{hnf} } {\mu_{f} }}} \right. \kern-0pt} {\mu_{f} }}}}{{{{\rho_{hnf} } \mathord{\left/ {\vphantom {{\rho_{hnf} } {\rho_{f} }}} \right. \kern-0pt} {\rho_{f} }}}} + \beta \xi^{2} \left( 2 \right)} \right)}}, $$

18 $$ \xi^{\prime}\left( 5 \right) = - \left\{ {\frac{{\frac{{{{\mu_{hnf} } \mathord{\left/ {\vphantom {{\mu_{hnf} } {\mu_{f} }}} \right. \kern-0pt} {\mu_{f} }}}}{{{{\left( {\rho C_{p} } \right)_{hnf} } \mathord{\left/ {\vphantom {{\left( {\rho C_{p} } \right)_{hnf} } {\left( {\rho C_{p} } \right)_{f} }}} \right. \kern-0pt} {\left( {\rho C_{p} } \right)_{f} }}}}\Pr Ec\xi^{2} \left( 3 \right) + \Pr \xi \left( 1 \right)\xi \left( 5 \right) + \frac{\Pr }{{{{\left( {\rho C_{p} } \right)_{hnf} } \mathord{\left/ {\vphantom {{\left( {\rho C_{p} } \right)_{hnf} } {\left( {\rho C_{p} } \right)_{f} }}} \right. \kern-0pt} {\left( {\rho C_{p} } \right)_{f} }}}}\left( {Nb\xi \left( 5 \right)\xi \left( 7 \right) + Nt\xi^{2} \left( 5 \right)} \right)}}{{\frac{{{{k_{hnf} } \mathord{\left/ {\vphantom {{k_{hnf} } {k_{f} }}} \right. \kern-0pt} {k_{f} }}}}{{{{\left( {\rho C_{p} } \right)_{hnf} } \mathord{\left/ {\vphantom {{\left( {\rho C_{p} } \right)_{hnf} } {\left( {\rho C_{p} } \right)_{f} }}} \right. \kern-0pt} {\left( {\rho C_{p} } \right)_{f} }}}}}}} \right\}, $$

19 $$ \xi^{\prime}\left( 7 \right) = - \left\{ {Sc\xi \left( 1 \right)\xi \left( 7 \right) + \frac{Nt}{{Nt}}\xi^{\prime}\left( 5 \right) - K_{r} Sc\xi \left( 6 \right)} \right\}, $$

20 $$ \left\{ \begin{aligned} \, & \xi_{a} \left( 1 \right) - S,\,\,\,\,\xi \left( 2 \right) - 1,\,\,\,\,\xi_{b} \left( 2 \right) - 0, \\ & \frac{{k_{hnf} }}{{k_{f} }}\xi \left( 5 \right) = Bi\left( {\xi \left( 4 \right) - 1} \right),\,\,\,\xi_{b} \left( 4 \right) - 0, \\ \, & Nb\xi \left( 7 \right) + Nt\xi \left( 5 \right) - 0,\,\,\,\,\xi \left( 6 \right) - 0. \\ \end{aligned} \right\} $$

## Validation

We validate our results for $\Pr$ with unlike values, when $\varphi_{{p_{1} }} = 0$, $\varphi_{{p_{2} }} = 0$, $M = 0$, $\beta = 0$, $Sc = 0$, $K_{r} = 0$, $Nt = 0$, $Ec = 0$, $Nb = 0$ and $Sc = 0$. Additionally, the convective condition is eliminated so the parameter $Bi$ vanished. Comparing our current findings with the results from the authors referenced in Table 2 shows a strong agreement, affirming the accuracy of our analysis. This validation is not only reaffirming the correctness of our methodology but also founds a strong continuity with recognized knowledge. Our new results and the published results further strengthen the credibility and reliability of our research outcomes.

**Table 2:** Comparison of our results with established work for varying values of $\Pr$ and keeping other factors as zeros.

$\Pr$	Khan and Pop^50^	Wang^51^	Gorla and Sidawi^52^	Devi and Devi^53^	Grubka and Bobba^54^	Gowda et al.^55^	Dawar et al.^45^	Present results
0.01	–	–	–	–	0.0099	0.00978	0.01566480561	0.01566480
0.07	0.0656	0.0656	0.0656	–	–		0.06562257644	0.06562257
0.2	0.1691	0.1691	0.1691	–	–		0.16908861990	0.16908861
0.7	0.4539	0.4539	0.4539	–	–		0.45391742750	0.45391742
0.72	–	–	–	–	0.4631	0.46273	0.46314470980	0.46314470
1.0	–	–	–	–	0.5820	0.58193	0.58197690060	0.58197690
2.0	0.9114	0.9114	0.9114	0.91135	–	–	0.91136139280	0.91136139
7.0	1.8954	1.8954	1.8905	–	–	–	1.89542020000	1.89542020

## Discussion of results

**Figure 2:**
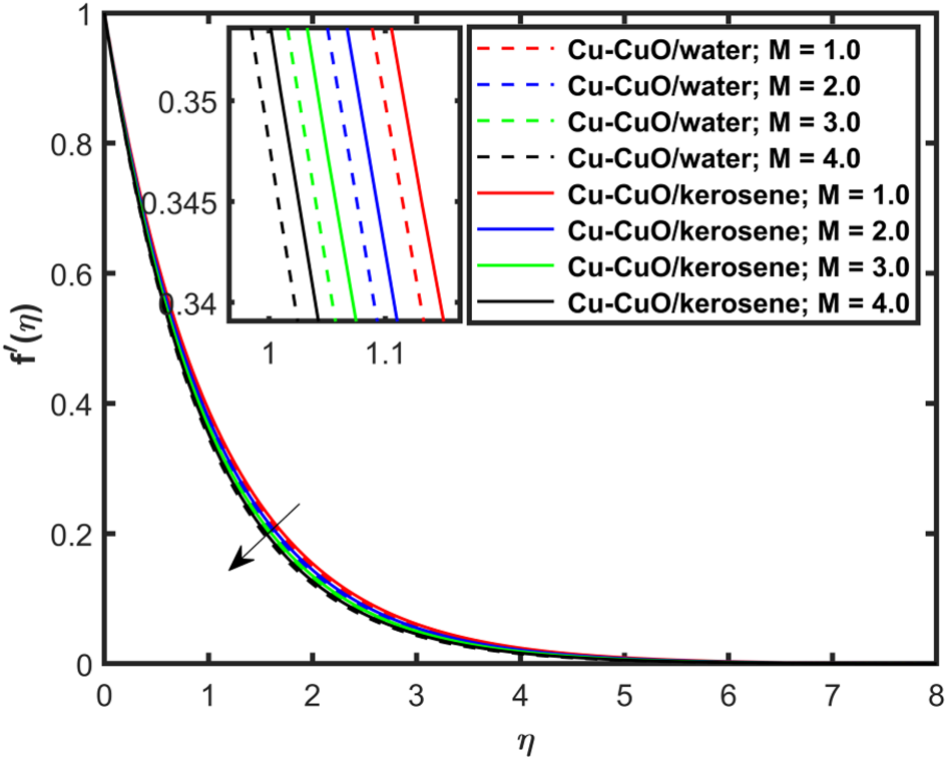
Impact of $M$ on $f^{\prime}\left( \eta \right)$.

**Figure 3:**
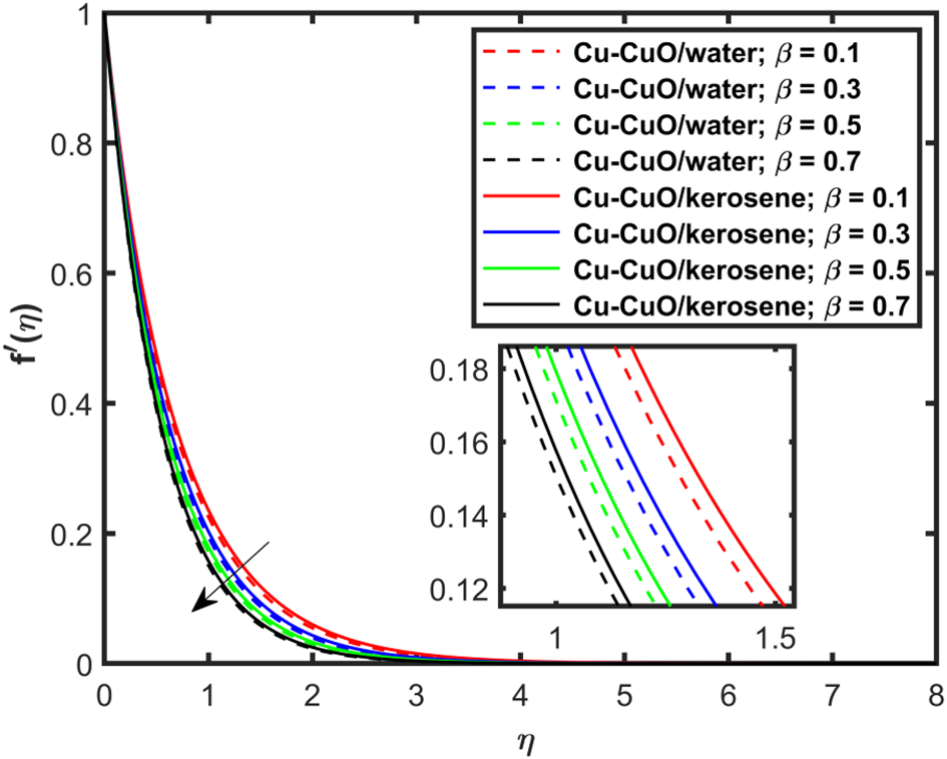
Impact of $\beta$ on $f^{\prime}\left( \eta \right)$.

**Figure 4:**
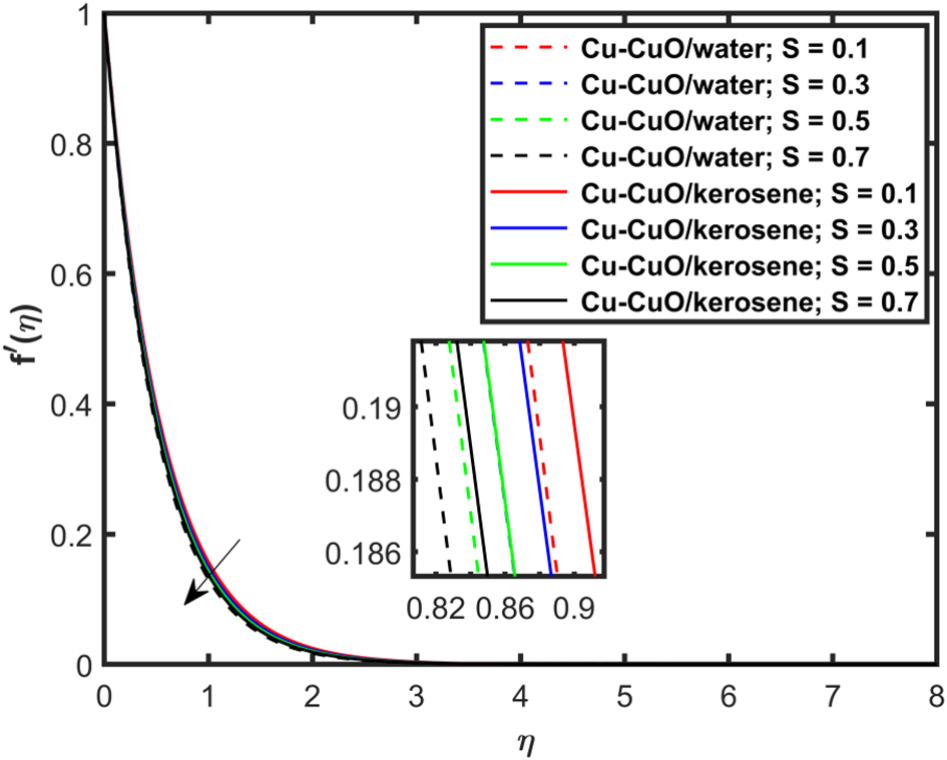
Impact of $S$ on $f^{\prime}\left( \eta \right)$.

**Figure 5:**
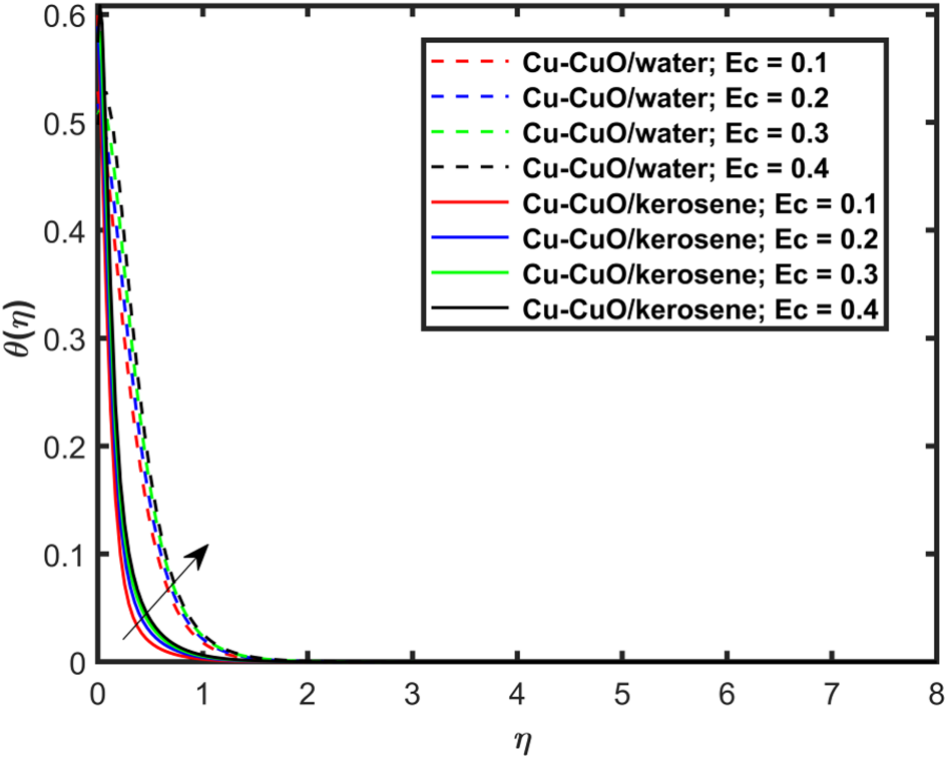
Impact of $Ec$ on $\theta \left( \eta \right)$.

**Figure 6:**
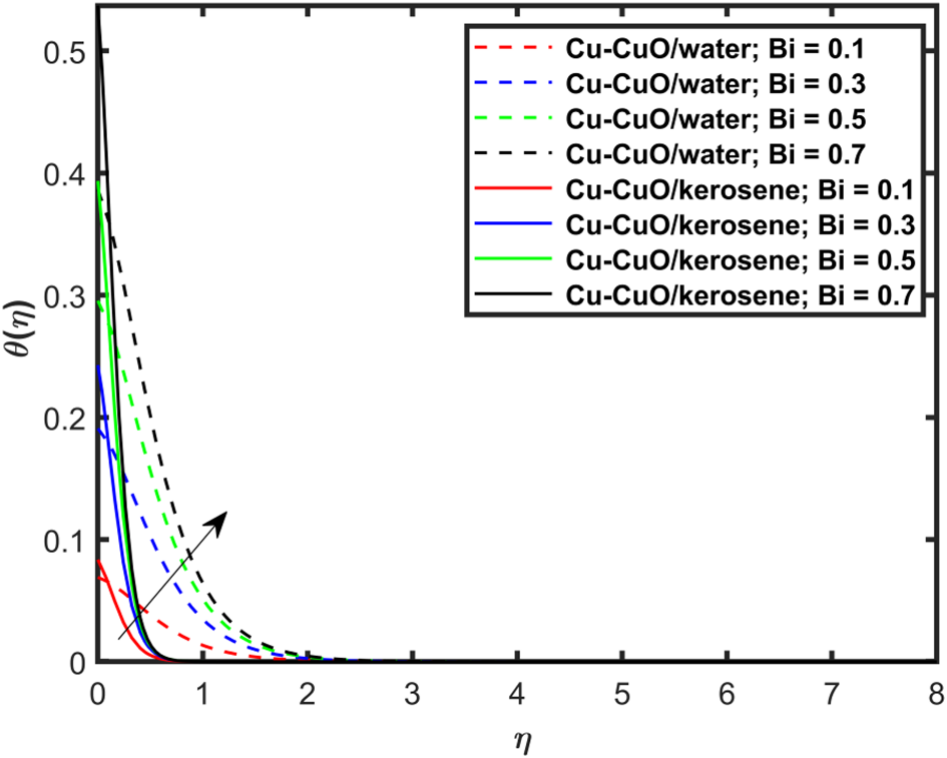
Impact of $Bi$ on $\theta \left( \eta \right)$.

**Figure 7:**
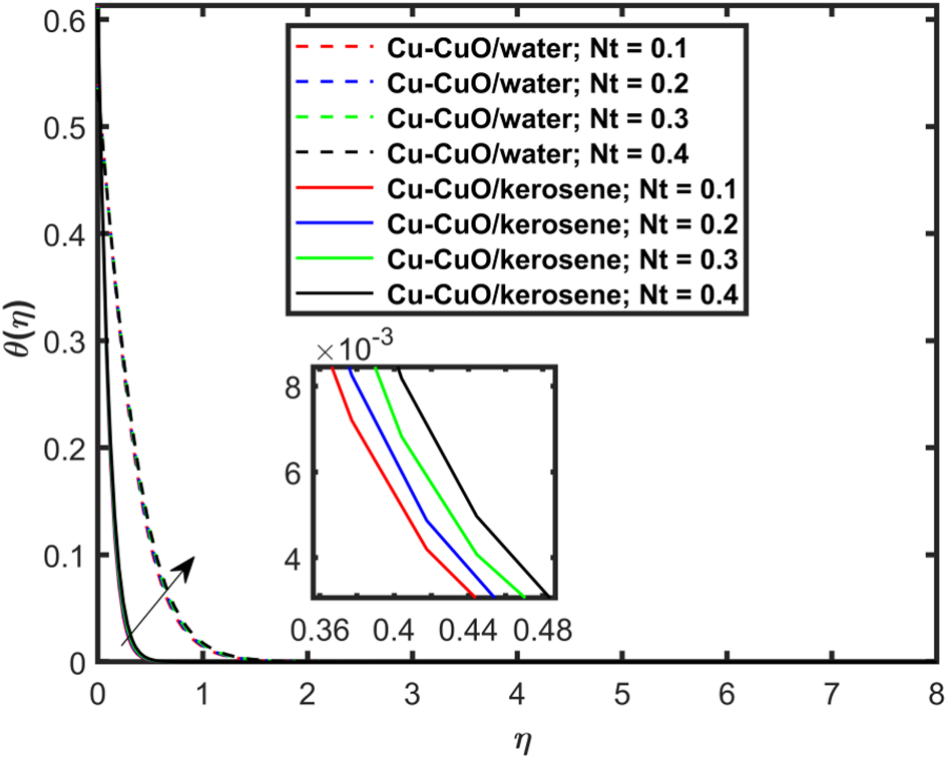
Impact of $Nt$ on $\theta \left( \eta \right)$.

**Figure 8:**
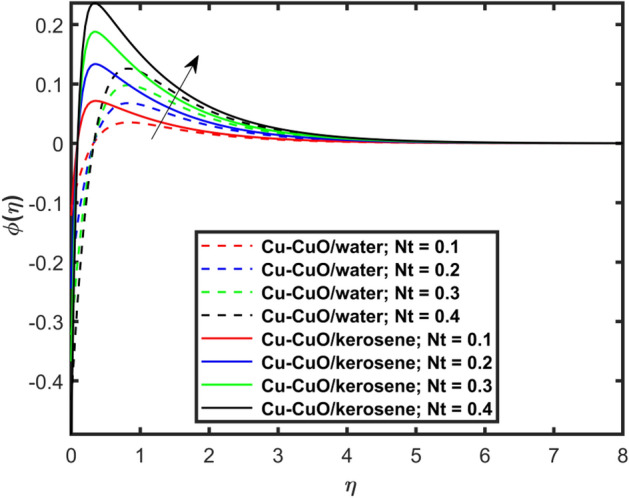
Impact of $Nt$ on $\phi \left( \eta \right)$.

**Figure 9:**
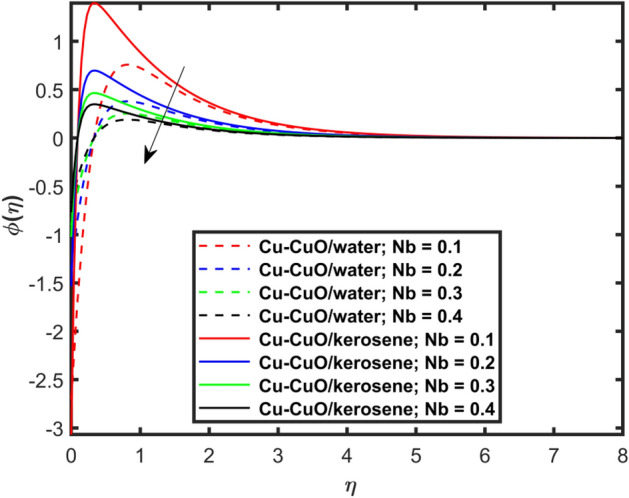
Impact of $Nb$ on $\phi \left( \eta \right)$.

**Figure 10:**
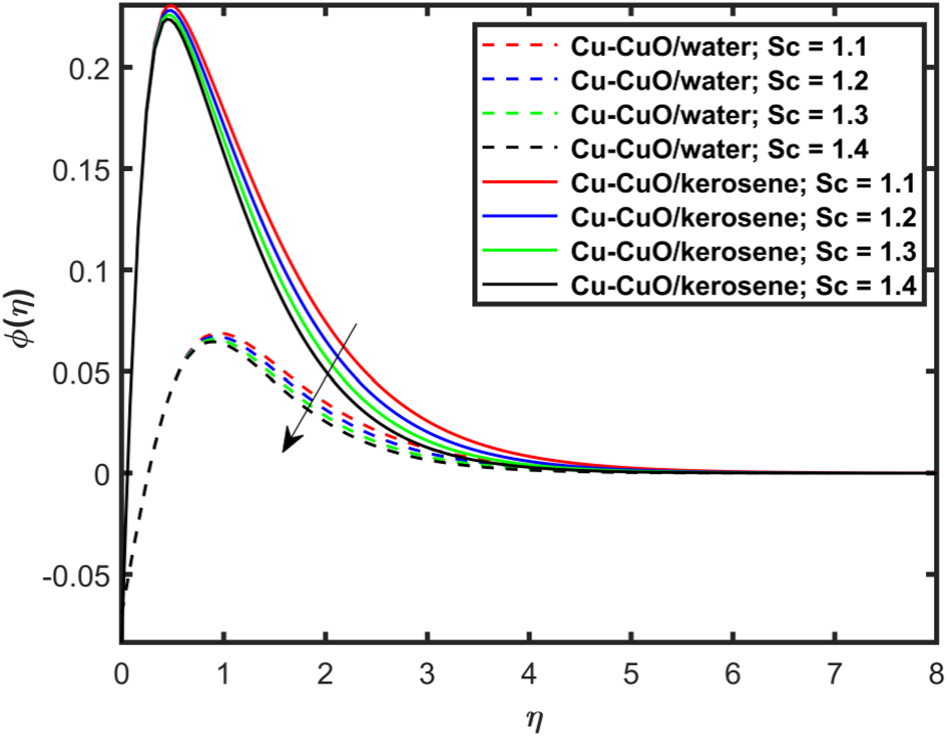
Effect of $Sc$ on $\phi \left( \eta \right)$.

**Figure 11:**
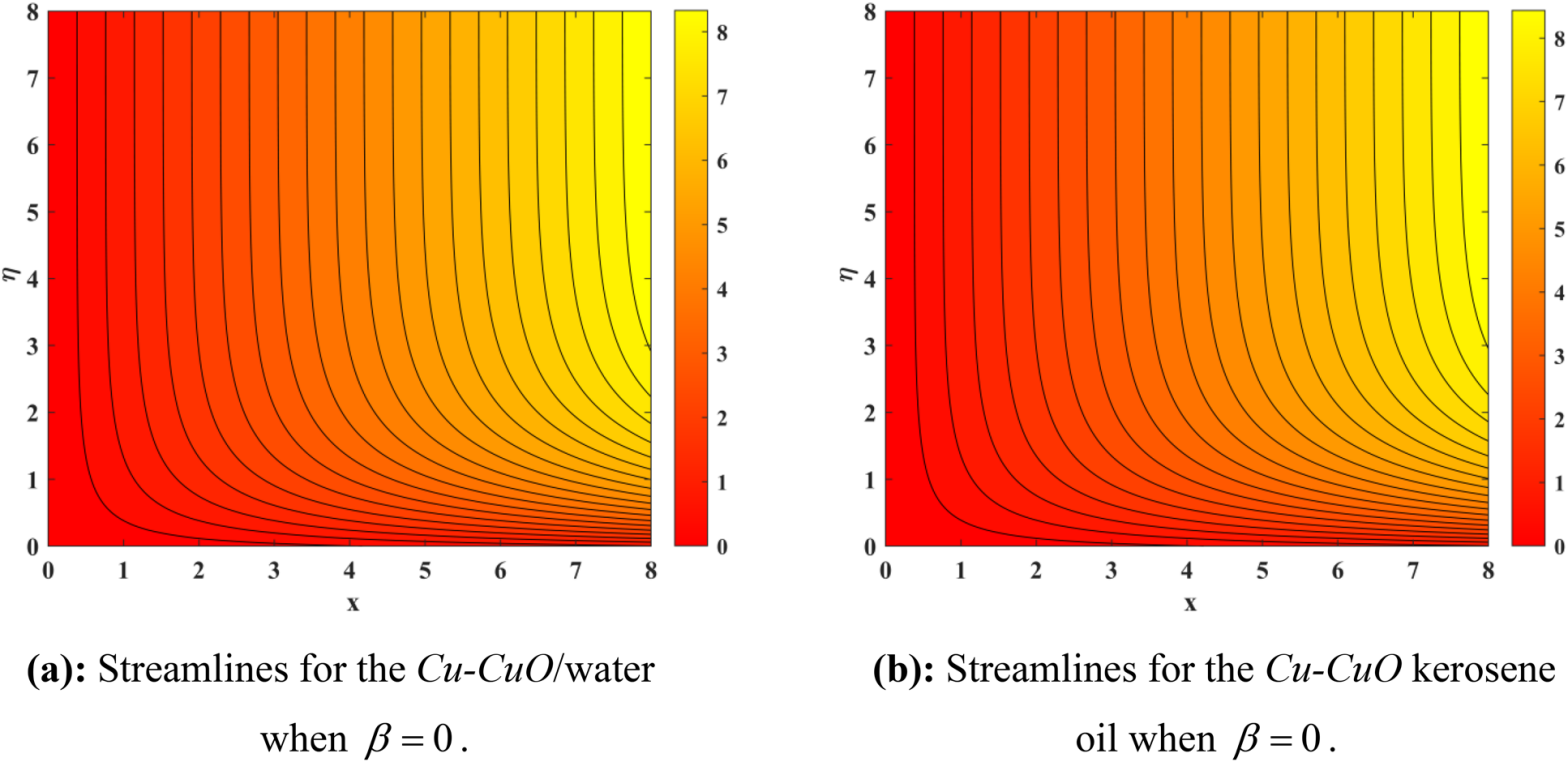
(**a**) Streamlines for the Cu–CuO /water when $\beta = 0$. (**b**) Streamlines for the Cu–CuO kerosene oil when $\beta = 0$.

**Figure 12:**
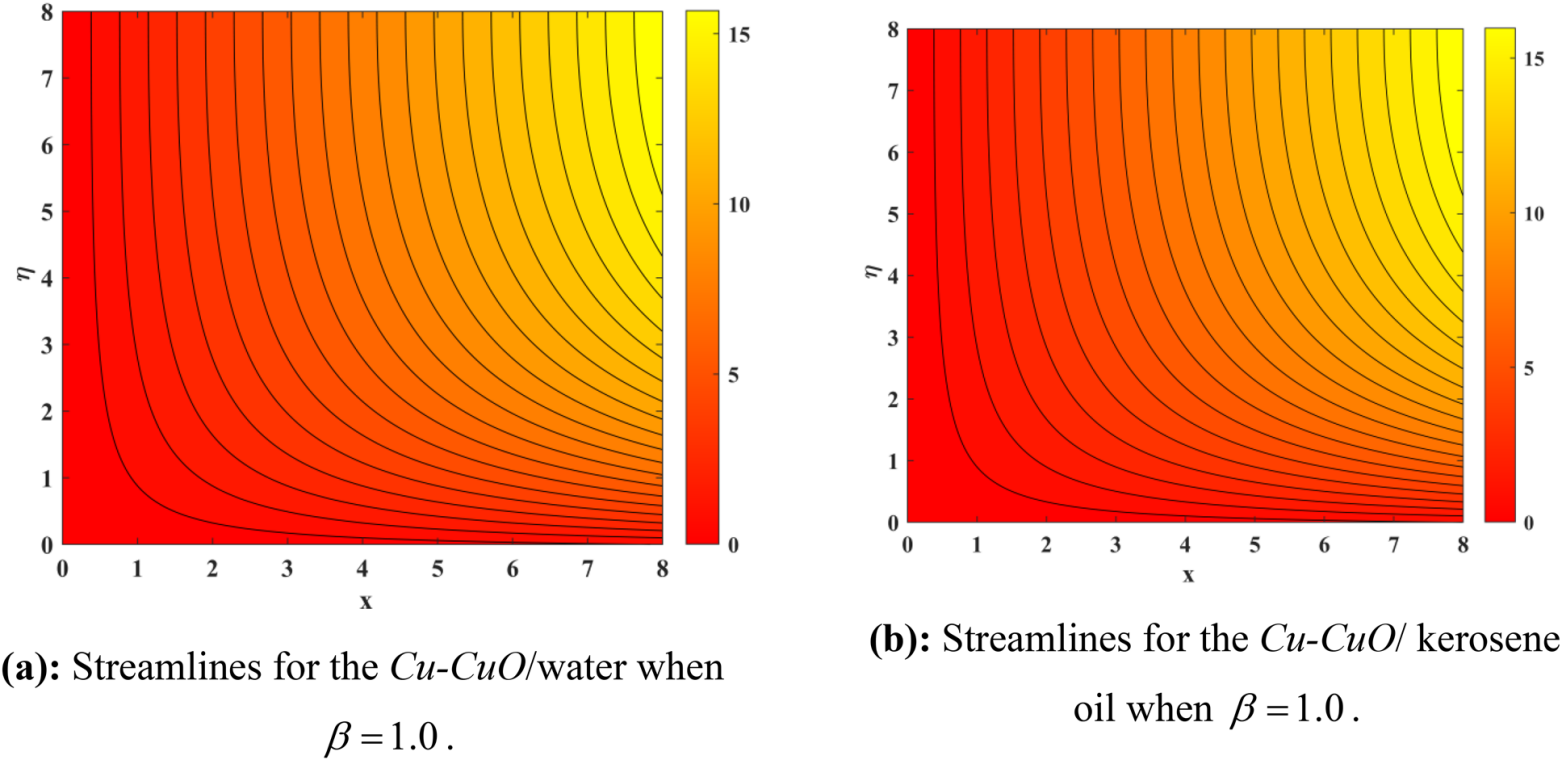
(**a**) Streamlines for the Cu–CuO /water when $\beta = 1.0$. (**b**) Streamlines for the Cu–CuO/kerosene oil when $\beta = 1.0$.

**Figure 13:**
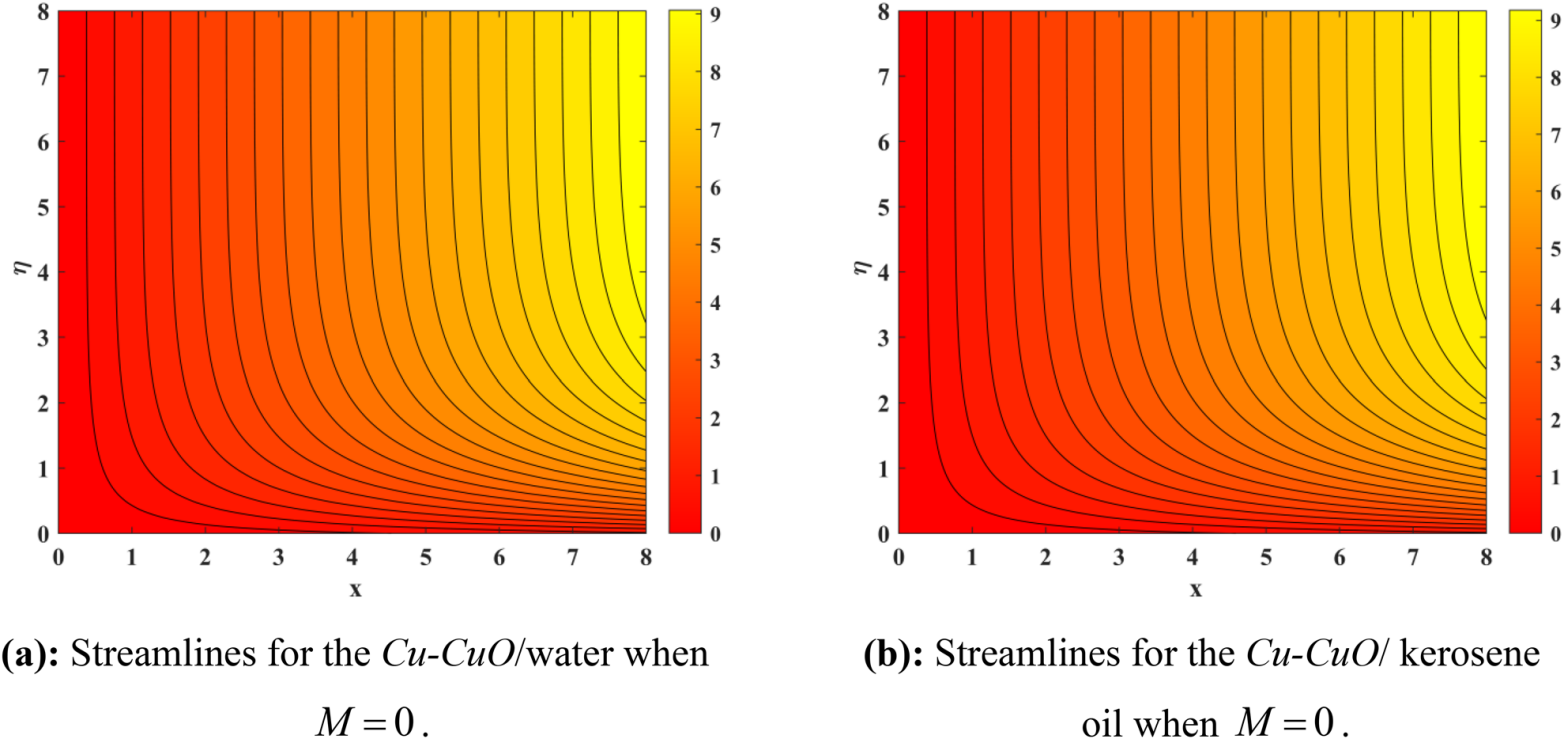
(**a**) Streamlines for the Cu–CuO /water when $M = 0$. (**b**) Streamlines for the Cu–CuO / kerosene oil when $M = 0$.

**Figure 14:**
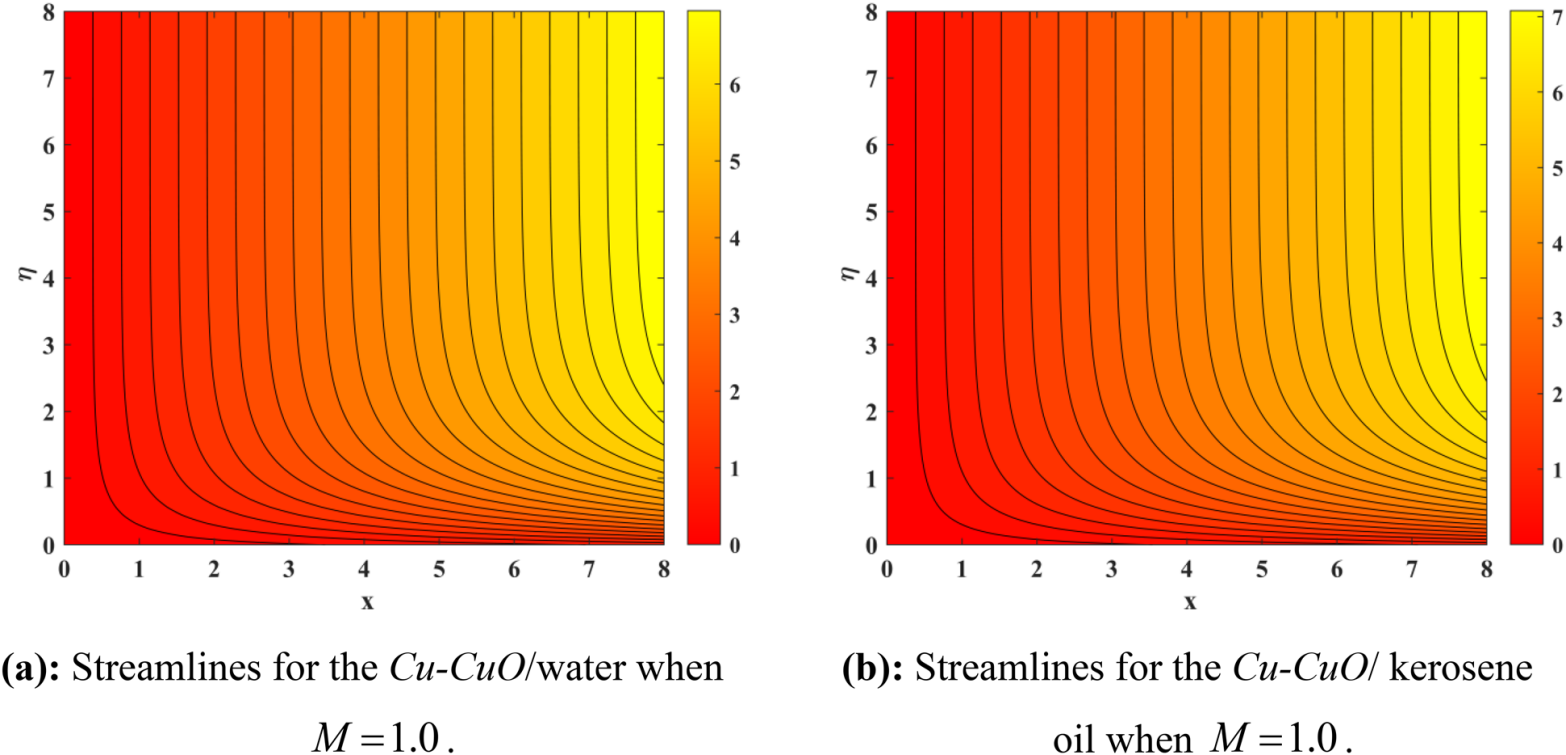
(**a**) Streamlines for the Cu–CuO /water when $M = 1.0$. (**b**) Streamlines for the Cu–CuO kerosene oil when $M = 1.0$.

This section explains the impacts of various physical factors on the velocity, temperature and concentration profiles are determined with the help of Figures. Furthermore, the impacts of embedded factors on skin friction and local Nusselt number are determined with the help of Tables. The impression of $M$ on ($f^{\prime}\left( \eta \right)$) is presented in Fig. 2. From this Figure, we observed that $f^{\prime}\left( \eta \right)$ reduces via increasing $M$. Physically, the magnetic factor is associated with a Lorentz force which always acts against the particles motion. When $M$ raises the Lorentz force supports and so as the opposing forces that results diminishing the fluid velocity with up-surging force of friction. This increasing friction force reduces $f^{\prime}\left( \eta \right)$. Here, two types of pure fluids have used in the work so, comparing both the base fluids, the kerosene oil-based hybrid nanofluid flow have higher velocity than that of water-based hybrid nanofluid flow. It depends on many physical properties of the base fluids like density, heat capacitance, viscosity, and electrical conductivity. The electrical conductivity of the water is higher than that of the kerosene oil because water has ions like sodium and chloride what makes it higher conductive. On the other hand, kerosene oil is typically a poor conductor of electricity. Also, the study of fluids over different physical surfaces also plays an important role. Since, water is more electrically conductive than that of kerosene oil, so the velocity profile of the water-based hybrid nanofluid flow will smaller that of kerosene oil when studying over a stretching surface. The reason is that the kerosene oil has less electrically conductive. By this discussion, we conclude that the kerosene oil-based fluid has higher velocity than that of water-based fluid. Effect of $\beta$ on $f^{\prime}\left( \eta \right)$ is exhibited in Fig. 3. The higher $\beta$ diminishes $f^{\prime}\left( \eta \right)$. Physically, the higher Deborah number ($0 < \beta < 1$), the viscous forces are dominant over an elastics forces and the fluid behave like a Newtonian one. As the viscous forces are higher for lower Deborah number, the hybrid nanofluids flows velocities reduce. Comparing the two different base fluids, the Deborah number, in general, has no relation with two considered base fluids. However, the magnetic field plays an important role as shown in Eq. (8) of proposed model. Hybrid nanofluid in case of kerosene oil has greater velocity than water-based fluid (hybrid) as discussed in Fig. 2. The impression of $S$ on $f^{\prime}\left( \eta \right)$ is exhibited in Fig. 4. From this figure it obvious that growth in $S$ reduces $f^{\prime}\left( \eta \right)$. The reason is that the increasing suction factor reduces width of momentum layer at the boundary which results reduction in $f^{\prime}\left( \eta \right)$. Additionally, it is found that the suction factor has greater velocity for kerosene oil-based nano-liquid (hybrid) in contrast of water-base fluid (hybrid nanofluid). The effects of $Ec$ on thermal distribution ($\theta \left( \eta \right)$) is exposed in Fig. 5. The higher $Ec$ increases $\theta \left( \eta \right)$. This number characterizes the relative importance of heat transmission to the kinetic energy. The reason is that the higher $Ec$ increases the kinetic energy of the hybrid nanofluid flow which results higher temperature boundary layer. As a results the temperature distribution augments $\theta \left( \eta \right)$. In comparison to kerosene oil, water has higher than conductivity. So, the temperature of the water-based hybrid nanofluid flow is obviously higher. Also, specific heat and density of the water and kerosene oil are other factors which also depends the heat transfer rates of the fluids. But the thermal conductance is the dominant reason for the present case. The impression of $Bi$ on $\theta \left( \eta \right)$ is displayed in Fig. 6. Escalation in $Bi$ is responsible for upsurge in $\theta \left( \eta \right)$. By definition, thermal Biot number is in direct relation with heat transfer coefficient which means that as we increase $Bi$, so as coefficient of thermal flow. In general, the fluid which has lower thermal conductivity will have greater temperature at the sheet’s surface. Since water has lesser thermal conductivity than that of kerosene oil, so hybrid nanofluid in case of water has greater temperature than kerosene oil based fluid (hybrid). Figure 7 depicts impression of $Nt$ on $\theta \left( \eta \right)$. It is perceived that the higher $Nt$ increases $\theta \left( \eta \right)$. A phenomenon in which the fluid particles migrate from high temperature region to lower temperature region is called thermophoresis. Thus, the higher temperature boundary layer thickness is perceived and so as temperature distribution. In comparison to kerosene oil, water has higher conductivity. So, the temperature of the water-based hybrid nanofluid flow is obviously greater. The impression of $Nt$ is demonstrated in Fig. 8 on $\phi \left( \eta \right)$. It perceived that higher $Nt$ increases $\phi \left( \eta \right)$. The greater $Nt$ increases the concentration boundary layer which results higher concentration profile. Hybrid nanofluid in case of water has greater concentration than kerosene oil based fluid (hybrid). The impact of $Nb$ on $\phi \left( \eta \right)$ is exhibited in Fig. 9. It is perceived that upsurge in $Nb$ reduces $\phi \left( \eta \right)$. Hybrid nanofluid in case of water has greater concentration than kerosene oil based fluid (hybrid). Figure 10 depicts impression of $Sc$ on $\phi \left( \eta \right)$ with a reducing trend in concentration. Physically, the kinematic viscosity increases and mass diffusivity reduces with the increasing $Sc$. This means that greater $Sc$ reduces the mass diffusivity which results reduction in the concentration profile. Comparing water and kerosene oil, water has higher mass diffusivity and lower kinematic viscosity which means that the water has greater mass diffusion compared to momentum transmission. On the other hand, kerosene oil has lower mass diffusivity and higher kinematic viscosity which means that kerosene oil has lower mass diffusion compared to momentum transmission. Combining these effects, water has smaller $Sc$ in comparison of kerosene oil. Hybrid nanofluid in case of water has lower concentration than kerosene oil based fluid (hybrid). Figure 11a and b show the streamlines of a water-based and kerosene-oil-based Newtonian hybrid nanofluids flows over a stretching surface. We know that the viscosity of water is lower than that of kerosene oil which means that the kerosene oil shows greater resistance to deformation than that of water. From these two Fig. 11a and b, we see that the streamlines of a kerosene oil-based hybrid nanofluid flow is more stretched than that of water-based hybrid nanofluid flow. The reason is that the kerosene oil shows greater resistance to deformation when compared to water. Figure 12a,b portrays the streamlines of fluid flow on stretching surface. As discussed in Fig. 11a and b that the kerosene oil has greater resistance to deformation than that of water, same is here. Actually, the value of non-Newtonian factor (Deborah number) is considered to be constant and equal in both Figures. Figure 13a and b show the streamlines of fluid flow on stretching surface when the flow is not magnetized. Again we have the same for the two different base fluids. So, the streamlines of the hybrid nanofluid based on kerosene are much closer than that of water-based nanofluid (hybrid). Figure 14a and b show the streamlines for both types of non-Newtonian hybrid nanofluids flow over a stretching surface when the flow is magnetized (magnetic field is considered). Again we that, the streamlines of the hybrid nanofluid based on kerosene are much closer than that of water-based nanofluid (hybrid). Additionally, here, we have considered the impact of magnetic field factor on the streamlines behaviors for the two hybrid nanofluids flows while considering two different base fluids. When comparing the two different base fluids, velocity is much higher for hybrid nanofluid based on kerosene oil than water-based nanofluid (hybrid) which means that there is more resistive force at the sheet’s surface for the flow of a kerosene oil-based hybrid nanofluid flow. Therefore, we have experienced that the streamlines of a kerosene oil-based hybrid nanofluid flow are closer to each other than that of water. Table 3 portrays impression of $M$ and $\beta$ on ${\text{Re}}_{x}^{1/2} C_{fx}$ for both types of fluid flow. From this Table, we observe that the increasing $M$ and $\beta$ increases ${\text{Re}}_{x}^{1/2} C_{fx}$ for both type of fluids. The physical reasons for each factor have been discussed in the relative Figures. Table 4 depicts the impacts of $Ec$, $Nt$ and $M$ on ${\text{Re}}_{x}^{ - 1/2} Nu_{x}$ for both types of fluid flow. From this Table, we observe that the increasing $Ec$, $Nt$ and $M$ increases ${\text{Re}}_{x}^{ - 1/2} Nu_{x}$ for both type of fluid. The physical reasons for each factor have been discussed in the relative Figures.

**Table 3:** Impacts of $M$ and $\beta$ on ${\text{Re}}_{x}^{1/2} C_{fx}$ for both water-based and kerosene oil-based hybrid nanofluids flows.

$M$	$\beta$	Water	Kerosene
0.1		1.445274326	1.277500956
0.2		1.485248773	1.312072588
0.3		1.523913496	1.345536623
0.4		1.561389179	1.377992858
	0.1	0.988653324	0.872959859
	0.2	1.119700222	0.988930075
	0.3	1.271039103	1.123016462
	0.4	1.445274326	1.277500956

**Table 4:** Impacts of $Ec$, $Nt$ and $M$ on ${\text{Re}}_{x}^{ - 1/2} Nu_{x}$ for both water-based and kerosene oil-based hybrid nanofluids flows.

$Ec$	$Nt$	$M$	Water	Kerosene
0.1			0.3779389807	0.4024913888
0.2			0.3982271383	0.4235701619
0.3			0.4185865224	0.4447244990
0.4			0.4390193899	0.4659723174
	0.1		0.4591429341	0.4870499559
	0.2		0.4593227192	0.4871194630
	0.3		0.4594971537	0.4871856880
	0.4		0.4596664899	0.4872488488
		1.0	0.4589481136	0.4869544393
		2.0	0.4590357871	0.4870250457
		3.0	0.4591720805	0.4871138057
		4.0	0.4592407210	0.4872147572

## Conclusion

Hybrid nanofluid flow on an extending surface that contains copper and copper oxide nanoparticles has been examined in this article. Kerosene oil and water considered as pure fluids. In this analysis, thermal convection and zero mass flux constraints are employed with impacts of magnetic, suction, chemical reaction, Brownian motion, and thermophoresis. This investigation compared the impacts of these variables on the hybrid nanofluid flow via stretching surface. After the present study is complete, the following observations are made:

- Velocity distribution weakened with upsurge in suction and magnetic variables. Hybrid nanofluid in case of kerosene oil has greater velocity than water-based fluid (hybrid).
- The higher Deborah number reduces the velocity profile. When compared the two different base fluids, it is found that the Deborah number, in general, has no relation to the two considered base fluids.
- The temperature profile increased due to the greater thermal Biot number, Eckert number, and thermophoresis factors. Hybrid nanofluid in case of water has greater temperature than kerosene oil based fluid (hybrid).
- Brownian motion factor and Schmidt number diminish the larger thermophoresis factor, which raises the concentration profiles. Hybrid nanofluid in case of water has greater concentration than kerosene oil based fluid (hybrid).
- Compared to both water-based Newtonian and non-Newtonian hybrid nanofluid flows, the streamlines of kerosene oil-based flows are more stretched.
- In comparison to the water-based hybrid nanofluid flow, the streamlines of the kerosene-oil-based hybrid nanofluid flow are closer to one another for both magnetised and non-magnetized flows.

### Future recommendations

It is recommended for future work to investigate nanofluids and hybrid nanofluid flows over an extending surface by considering different base fluids and nanoparticles and compare the obtained results with past results to get a collective conclusion. For example, the flow of sodium alginate-based hybrid nanofluid containing Cu and CuO will give different results from this study, and one can conclude the most effective results. With the same assumptions, one can also consider the thermal convective and zero-mass flux conditions.

## Data Availability

All data used in this manuscript have been presented within the article.

## List of symbols

$$u,v$$: Velocity components
$$x,y$$: Coordinate axes
$$u_{w}$$: Stretching velocity
$$c$$: Constant
$B_{0}$: Strength of magnetic field
$T_{f}$, $T_{w}$, $T_{\infty }$: Hot working fluid, Surface, Ambient temperatures
$C_{w}$, $C_{\infty }$: Surface, Ambient concentrations
$\lambda$: Relaxation time factor
$\mu$, $\nu$: Dynamic, kinematic viscosities
$$\rho$$: Density
$$\sigma$$: Electrical conductivity
$$k$$: Thermal conductivity
$$C_{p}$$: Specific heat
$D_{B}$, $D_{T}$: Coefficients of Brownian and thermophoresis diffusions
$Kr$: Chemical reaction coefficient
$\varphi$: Nanoparticle volume fraction
$$M$$: Magnetic factor
$$\beta$$: Deborah number
$$\Pr$$: Prandtl number
$$Ec$$: Eckert number
$Nb$, $Nt$: Brownian motion, thermophoresis factors
$K_{r}$: Chemical reaction factor
$Bi$: Thermal Biot number
$$S$$: Suction factor

## Subscripts

$f$, $nf$, $hnf$: Fluid, Nanofluid, Hybrid nanofluid
$$p_{1}$$: Firs nanoparticle
$$p_{2}$$: Second nanoparticle
